# High-throughput interspecies profiling of acidic plant hormones using miniaturised sample processing

**DOI:** 10.1186/s13007-022-00954-3

**Published:** 2022-11-16

**Authors:** Jitka Široká, Federica Brunoni, Aleš Pěnčík, Václav Mik, Asta Žukauskaitė, Miroslav Strnad, Ondřej Novák, Kristýna Floková

**Affiliations:** 1 Laboratory of Growth Regulators, Institute of Experimental Botany of the Czech Academy of Sciences & Palacký University, Šlechtitelů 27, 78371 Olomouc, Czech Republic; 2 Department of Experimental Biology, Faculty of Science, Palacký University, Šlechtitelů 27, 78371 Olomouc, Czech Republic; 3 Department of Chemical Biology, Faculty of Science, Palacký University, Šlechtitelů 27, 78371 Olomouc, Czech Republic; 4 Plant Hormone Biology Group, Swammerdam Institute for Life Sciences, University of Amsterdam, Science Park 904, 1098 XH Amsterdam, The Netherlands

**Keywords:** Plant hormones, Miniaturisation, In-tip microSPE, High-throughput, 3D printing, Liquid chromatography, Mass spectrometry, Evolutionarily distant plant species

## Abstract

**Background:**

Acidic phytohormones are small molecules controlling many physiological functions in plants. A comprehensive picture of their profiles including the active forms, precursors and metabolites provides an important insight into ongoing physiological processes and is essential for many biological studies performed on plants.

**Results:**

A high-throughput sample preparation method for liquid chromatography–tandem mass spectrometry determination of 25 acidic phytohormones classed as auxins, jasmonates, abscisates and salicylic acid was optimised. The method uses a small amount of plant tissue (less than 10 mg fresh weight) and acidic extraction in 1 mol/L formic acid in 10% aqueous methanol followed by miniaturised purification on reverse phase sorbent accommodated in pipette tips organised in a 3D printed 96-place interface, capable of processing 192 samples in one run. The method was evaluated in terms of process efficiency, recovery and matrix effects as well as establishing validation parameters such as accuracy and precision. The applicability of the method in relation to the amounts of sample collected from distantly related plant species was evaluated and the results for phytohormone profiles are discussed in the context of literature reports.

**Conclusion:**

The method developed enables high-throughput profiling of acidic phytohormones with minute amounts of plant material, and it is suitable for large scale interspecies studies.

**Supplementary Information:**

The online version contains supplementary material available at 10.1186/s13007-022-00954-3.

## Background

The acidic phytohormones are structurally diverse signalling molecules regulating plant growth, development and defence. They comprise the phytohormonal classes of auxins (AUXs), jasmonates (JAs), abscisates (ABAs), salicylates (SAs) and gibberellins. AUXs are indolic compounds, precursors and metabolites of bioactive indole-3-acetic acid (IAA), which is crucial to the regulation of almost every aspect of growth and development throughout a plant life [1]. The catabolism of IAA can happen either reversibly through the formation of indole-3-acetyl-1-*O*-ß-D-glucose (IAA-glc), indole-3-acetyl aspartate (IAA-Asp) and indole-3-acetyl glutamate (IAA-Glu) [2] or irreversibly through the formation of 2-oxindole-3-acetic acid (oxIAA) [3] and, in turn, oxIAA can be further glycosylated to 2-oxindole-3-acetyl-1-*O*-ß-D-glucose (oxIAA-glc) [4–6]. The IAA conjugation and oxidation mechanisms appeared early during plant evolution and are evolutionarily conserved across the whole plant kingdom [7–10]. JAs are cyclopentenones or cyclopentanones induced by mechanical stresses or responding to wounding or necrotrophic pathogen and herbivore attacks and they contribute to the regulation of plant growth and developmental processes [11]. The pathway for jasmonate biosynthesis begins with the conversion of α-linolenic and roughanic acid to *cis*-( +)-12-oxo-phytodienoic acid (*cis-*OPDA) and dinor-oxo-phytodienoic acid (dn-OPDA) respectively. These two cyclopentenone-ring-containing molecules are subsequently subjected to reduction and β-oxidation reactions that lead to the formation of jasmonic acid (JA). JA can be conjugated to amino acids to produce the biologically active form ( +)-7-*iso*-jasmonyl-L-isoleucine (JA-Ile) [12] or hydroxylated to inactive 12-hydroxyjasmonic acid (12-OHJA) (reviewed in [13]). While jasmonates are widely distributed in seed plants, the presence of JA and its derivatives in lower plants and algae is less understood, as the evidence regarding function of JAs in extant plant species is contradictory (reviewed in [14]). ABAs are isoprenoids derived from abscisic acid (ABA), which is responsive to abiotic stresses such as drought, salinity, heat and cold [15]. There are three different ABA hydroxylation pathways which oxidise one of the methyl groups of the ring structure (C-7′, C-8′, and C-9′), and the hydroxylation of ABA triggers further inactivation steps (reviewed in [16]). Hydroxylation at the C-8′ position is commonly thought to be the predominant ABA catabolic pathway. Consistent with this, the hydroxylated compounds phaseic acid (PA) and dihydrophaseic acid (DPA) are the most widespread and abundant ABA catabolites [16]. The 7′-hydroxyABA (7´-OHABA) form is found in a variety of plant species as a minor catabolite and 9’-hydroxyABA and its isomer neophaseic acid (neoPA) have been identified in several angiosperms [16]. SAs are phenolic compounds that have emerged as key plant defence molecules with critical roles in different aspects of plant immunity (reviewed in [17]). Despite the importance of salicylic acid (SA) in plant defence, its metabolic and signalling pathways are not fully understood. This is mainly due to the fact that these phenolic compounds have been relegated for a long time to the category of ‘secondary metabolites’ as they were considered non-essential for critical processes [18, 19]. Gibberellins are diterpenoids involved in plant developmental processes such as organ growth, seed germination, maturation and flowering [20]. Due to sensitivity issues caused by extremely low endogenous concentrations in plants and lower ionization efficiency of their molecules using mass spectrometry (MS) detection [21–23], they were omitted from our method.

The endogenous levels of the signalling forms of hormones and hence their activities in plants are tightly modulated by a combination of biosynthesis, metabolism, transport or release from their conjugated forms [16, 24–26]. Data on their concentrations reflect processes occurring in plants and offer an important insight into plant physiology. Depending on the plant tissue or the plant species itself, phytohormones usually occur at very low concentrations. The analytical methods employed in the determination of plant hormone levels therefore need to be very sensitive and capable of covering wide ranges of concentrations in order to track both low basal levels and levels that are elevated in response to stimuli. Moreover, the plant matrix is quite challenging from the analytical point of view. Nowadays plant hormone quantification is mostly performed by means of liquid chromatography–tandem mass spectrometry (LC–MS/MS) systems with electrospray ionisation (ESI). The current trend is to analyse large numbers of phytohormones in decreasing amounts of plant material using simple, high-throughput sample preparation techniques to provide comprehensive information about phytohormonal levels with sufficient statistical significance.

Due to the different chemical natures of targeted analytes, sample preparation from plant material prior to multi-hormone LC–MS/MS analysis is based on one of two strategies: simple, more generic, or laborious, comprising several selective steps. The simplest way is to only employ an extraction step [27–30]. This approach usually requires larger amounts of plant material, hundreds of mg fresh weight (FW) or tens of mg dry weight (DW), reaching compromise between extraction efficiency and ion suppression caused by interfering matrix present in the extracted sample. Another approach is extraction combined with derivatisation, which selectively enhances ionisation of the targeted analytes and lowers the limits of MS detection in the presence of plant matrix. For this approach very small amounts (units of mg and lower) of plant tissue are sufficient [31]. The most common form of sample preparation is extraction followed by solid phase extraction (SPE) purification using ion exchange or reverse phase sorbents [32–34], which can be also carried out in a 96-well set up [35]. SPE purification reduces the amount of interfering matrix in the sample and enables pre-concentration of the analytes, and it usually requires tens of mg FW plant tissue.

Small-scale purification based on classical SPE takes advantage of sensitive MS/MS detection and the ability to pre-concentrate the extracted analytes, simultaneously reducing the consumption of sample, sorbents and solvents [36, 37]. Miniaturisation also facilitates automation, increases throughput in sample preparation and decreases the economic and environmental impact. The in-tip microSPE approach using small plates of Empore discs placed in ordinary pipette tips, originally developed for the purification of proteins [38], has been applied to the purification of phytohormone classes such as cytokinins [39] and AUXs [40] in units of mg FW *Arabidopsis thaliana* plant tissue.

The aim of this work was to develop and validate a simple, miniaturised, high-throughput method for acidic phytohormone profiling from small amounts of plant samples that would be widely applicable in large-scale interspecies studies. Here we use this approach to analyse different amounts of nine matrices from plant species ranging from algae to land plants. The selected plant species are models often used in plant research. Moreover, they were chosen with respect to the representation of various groups within the phylogenetic tree. Determination of endogenous phytohormone profiles from evolutionarily related landmark species provides further substantial tiles to identify patterns that may have contributed to the diversification of extant plant lineages.

## Results and discussion

### LC–MS/MS optimisation

**Fig. 1:**
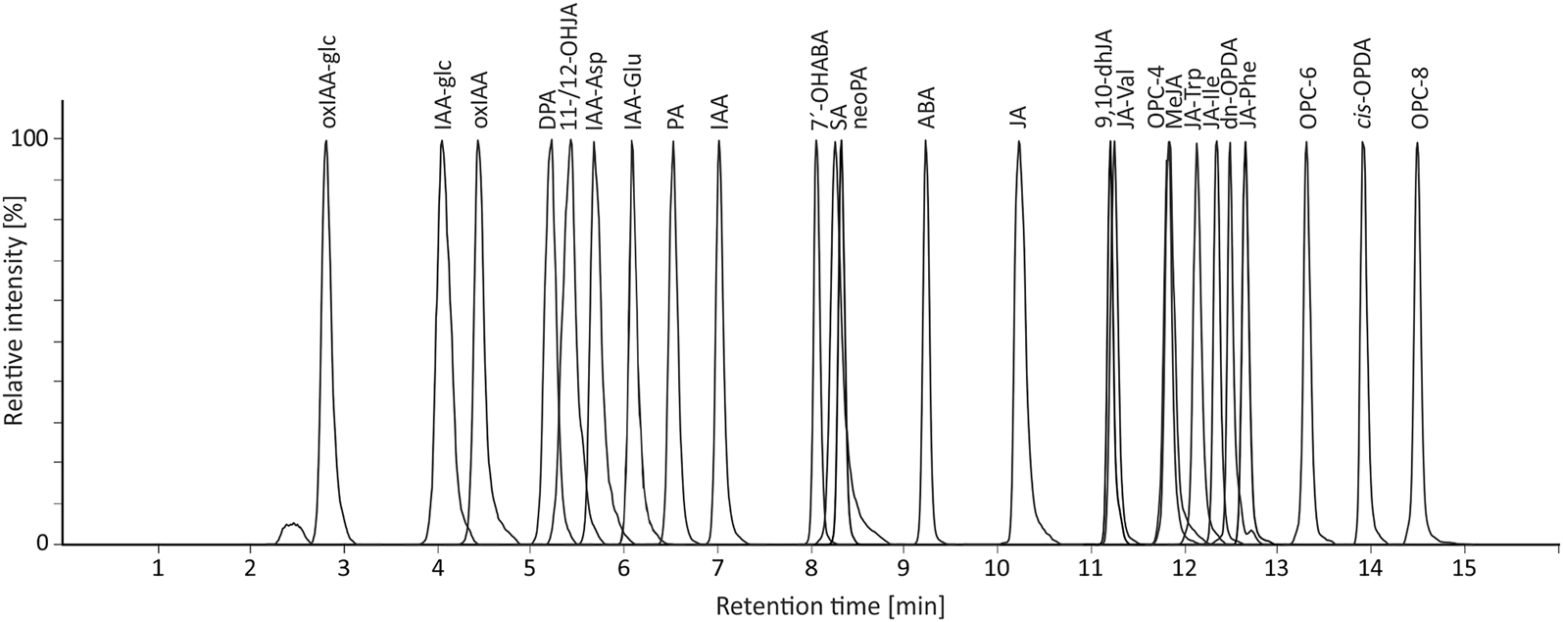
LC–MS/MS analysis of 25 acidic phytohormones, their precursors and metabolites

LC–MS analysis of plant hormones predominantly employs reverse stationary phases, particularly columns with C18 chemistry [28, 29, 31, 32, 34, 35]. To retain acidic phytohormones, taking into account the requirements for MS detection and the type of stationary phase, the mobile phases typically consist of volatile additives (formic or acetic acid) in water and acidified or pure methanol or acetonitrile. In this study, to optimise separation conditions two reverse phase C18 columns were tested: UPLC CSH C18 (2.1 ×100 mm, 1.7 μm, Waters) and Kinetex Evo C18 (2.1 ×150 mm, 2.6 μm, Phenomenex). The analysis performed on the CSH column (1.7 μm particle size) under conditions using 10 mmol/L formic acid and acetonitrile as mobile phases [33] generated backpressures close to the limits of the 1290 Infinity LC system (Agilent), and the elution of polar analytes was concentrated at the very beginning of the linear gradient. Using a Kinetex Evo column with a larger particle size (2.6 μm) lowered the system backpressure, and methanol instead of acetonitrile increased retention of the most polar compounds (e.g. oxIAA-glc), improved the overall chromatographic separation of the analytes and shortened the analytical run to 20 min (Table 1, Fig. 1).

**Table 1:** LC–MS/MS method parameters

Analyte	Abbreviation	Multiple reaction monitoring transitions^a^	Collision energy, eV^a^	Ionisation	Retention time, min ^b^	Internal standard	Limit of detection, fmol ^c^	Linear range, pmol	p*K*_a_^d^
indole-3-acetic acid	IAA	176.1–130.1	24	[M + H]^+^	6.85 ± 0.01	[^13^C_6_]-IAA	0.5	0.005-50	4.66
2-oxindole-3-acetic acid	oxIAA	192.1–146.0	12	[M + H]^+^	4.34 ± 0.01	[^13^C_6_]-oxIAA	10	0.05–50	3.74
indole-3-acetyl aspartate	IAA-Asp	291.1–130.1	36	[M + H]^+^	5.54 ± 0.01	[^13^C_6_]-IAA-Asp	25	0.05–50	3.74
indole-3-acetyl glutamate	IAA-Glu	305.2–130.1	24	[M + H]^+^	5.94 ± 0.01	[^13^C_6_]-IAA-Glu	2.5	0.005—50	3.65
indole-3-acetyl-1-*O*-ß-D-glucose	IAA-glc	336.1–174.0	8	[M - H]^−^	4.02 ± 0.02	[^13^C_6_]-IAA-glc	10	0.05–50	5.10
2-oxindole-3-acetyl-1-*O*-ß-D-glucose	oxIAA-glc	352.2–190.0	8	[M - H]^−^	2.66 ± 0.02	[^13^C_6_]-oxIAA-glc	1	0.005–50	NP
*cis*-( +)-12-oxo-phytodienoic acid	*cis*-OPDA	293.2–275.2	12	[M + H]^+^	13.80 ± 0.01	[^2^H_5_]-OPDA	2.5	0.005–5	4.78
(*Z*)-8-[3-oxo-2-(pent-2-enyl)cyclopentyl]octanoic acid	OPC-8	295.2–135.0	20	[M + H]^+^	14.36 ± 0.01	[^2^H_5_]-OPDA	25	0.05–5	4.72
(*Z*)-6-[3-oxo-2-(pent-2-enyl)cyclopentyl]hexanoic acid	OPC-6	267.1 – 135.0	28	[M + H]^+^	13.18 ± 0.01	[^2^H_5_]-OPDA	25	0.05–5	4.65
(Z)-4-[3-oxo-2-(pent-2-enyl)cyclopentyl]butanoic acid	OPC-4	237.2 – 58.8	20	[M - H]^−^	11.71 ± 0.01	[^2^H_6_]-JA	25	0.05–50	4.55
dinor-oxo-phytodienoic acid	dn-OPDA	265.2 – 247.1	4	[M + H]^+^	12.51 ± 0.01	[^2^H_2_]-JA-Ile	25	0.05–5	4.60
(−)-jasmonic acid	JA	209.2–58.8	8	[M - H]^−^	10.08 ± 0.01	[^2^H_6_]-JA	0.75	0.005–50	4.71
(±)-methyl jasmonate	MeJA	225.3 – 151.2	12	[M + H]^+^	11.71 ± 0.01	[^2^H_6_]-MeJA	2.5	0.005–5	NP
(±)-9,10-dihydrojasmonic acid	9,10-dhJA	211.2–58.8	16	[M - H]^−^	11.09 ± 0.01	[^2^H_6_]-JA	2.5	0.005-50	4.77
11-hydroxyjasmonic acid/ 12-hydroxyjasmonic acid	11-/12-OHJA	225.1–59.0	8	[M - H]^−^	5.13 ± 0.03	[^2^H_6_]-JA	5	0.05–50	4.46
(−)-jasmonyl-L-valine	JA-Val	310.3–151.3	16	[M + H]^+^	11.60 ± 0.01	[^2^H_2_]-JA-Ile	1	0.005–5	4.01
(−)-jasmonyl-L-isoleucine	JA-Ile	324.3 – 151.2	16	[M + H]^+^	12.22 ± 0.01	[^2^H_2_]-JA-Ile	2.5	0.005–5	4.06
(−)-jasmonyl-L-tryptophan	JA-Trp	397.3–351.3	12	[M + H]^+^	12.00 ± 0.01	[^2^H_2_]-JA-Ile	50	0.1–5	3.39
(−)-jasmonyl-L-phenylalanine	JA-Phe	358.8–151.2	16	[M + H]^+^	12.52 ± 0.01	[^2^H_2_]-JA-Ile	2.5	0.005–4.5	3.97
salicylic acid	SA	137.1–92.8	16	[M − H]^−^	8.03 ± 0.03	[^2^H_4_]-SA	25	0.05–50	2.79
(+)-*cis,trans*-abscisic acid	ABA	263.2–153.1	8	[M − H]^−^	9.10 ± 0.01	[^2^H_6_]-ABA	0.25	0.005–50	4.50
phaseic acid	PA	279.1–205.1	12	[M − H]^−^	6.61 ± 0.01	[^2^H_3_]-PA	5	0.01–50	4.30
dihydrophaseic acid	DPA	281.2–237.1	8	[M − H]^−^	4.96 ± 0.02	[^2^H_3_]-DPA	25	0.05–50	4.34
neophaseic acid	neoPA	279.1–205.1	12	[M − H]^−^	8.41 ± 0.00	[^2^H_3_]-neoPA	1	0.005–50	4.30
7'-hydroxyabscisic acid	7'-OHABA	279.1–151.1	12	[M − H]^−^	7.90 ± 0.01	[^2^H_4_]-7'-OHABA	5	0.01–50	4.37

NP not predicted

^a^optimised using chemical standards

^b^means ± standard deviation (SD) (n = 5)

^c^limit of detection S/N > 3, expressed as amount of substance injected

^d^predicted p*K*_a_ in chemicalize.com

Using the optimised chromatographic method was not possible to distinguish between 11-OHJA and 12-OHJA, because both compounds have the same retention times and fragmentation pattern. As in [33], the sum of unresolved 11-OHJA and 12-OHJA has been used in this study. The 11-OHJA and 12-OHJA can be separated by a gas chromatography MS method [41] or by using LC–MS with isocratic elution (Acquity UPLC BEH C18 column, 92% of 0.1% formic acid in water and 8% of 0.1% formic acid acetonitrile) [42].

### High-throughput protocol optimisation

**Fig. 2:**
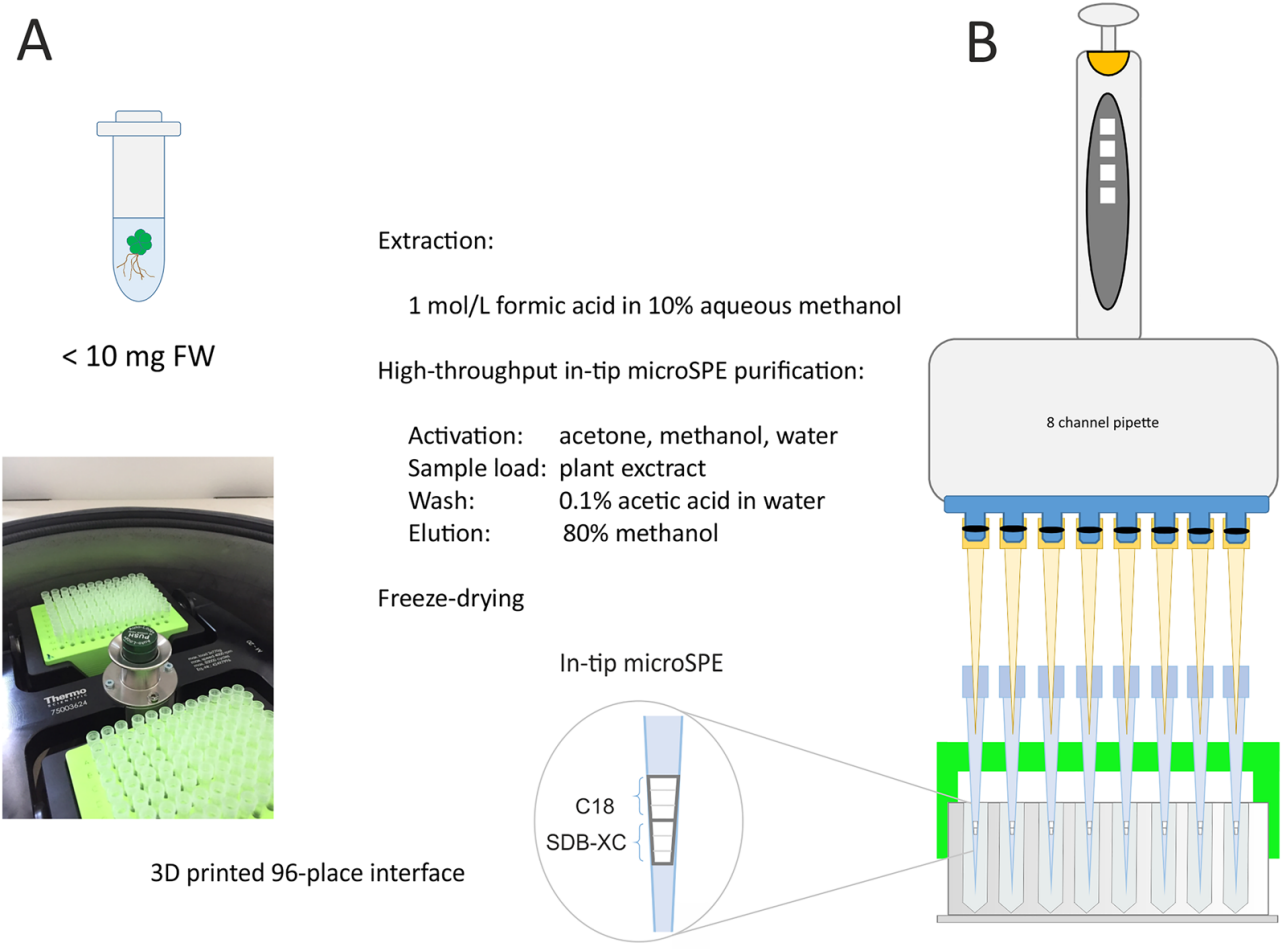
High-throughput sample preparation workflow

An in-tip microSPE stagetip assembled from SDB-XC (poly(styrenedivinylbenzene)) and C18 sorbents (Fig. 2) was used to optimise sample preparation conditions. Type and amount of the sorbent were set as previously published for AUXs [40]. Both microSPEs made up of SDB-XC/C18 and SPE Oasis® HLB (poly(divinylbenzene-co–*N*-vinylpyrrolidone) copolymer) sorbents provide reverse phase retention of analytes. The predicted p*K*_a_ values for acidic phytohormones range from 2.8 to 5.1 (Table 1). In order to improve their retention on reverse phase sorbents, analytes need to be in an uncharged form at a pH below their p*K*_a_, which in this case requires acidic conditions. This corresponds to the following SPE purification methods for individual acidic phytohormone classes. For auxin analysis, plant extracts in sodium phosphate buffer (50 mmol/L, pH 7) were adjusted to pH 2.7 with hydrochloric acid before loading on an Oasis^®^ HLB SPE column [43]. A similar approach was chosen for auxin metabolite profiling using a microSPE made from SDB-XC and C18 sorbents [40]. Purification of stress hormones (JAs, ABA, SA and IAA) on an Oasis^®^ HLB SPE column included a preconditioning step with 0.1% formic acid in water before loading plant extracts in 10% aqueous methanol [33]. ABA and its metabolites were extracted in 10% aqueous methanol containing 1% acetic acid before being purified on an Oasis^®^ HLB SPE column [44].

**Fig. 3:**
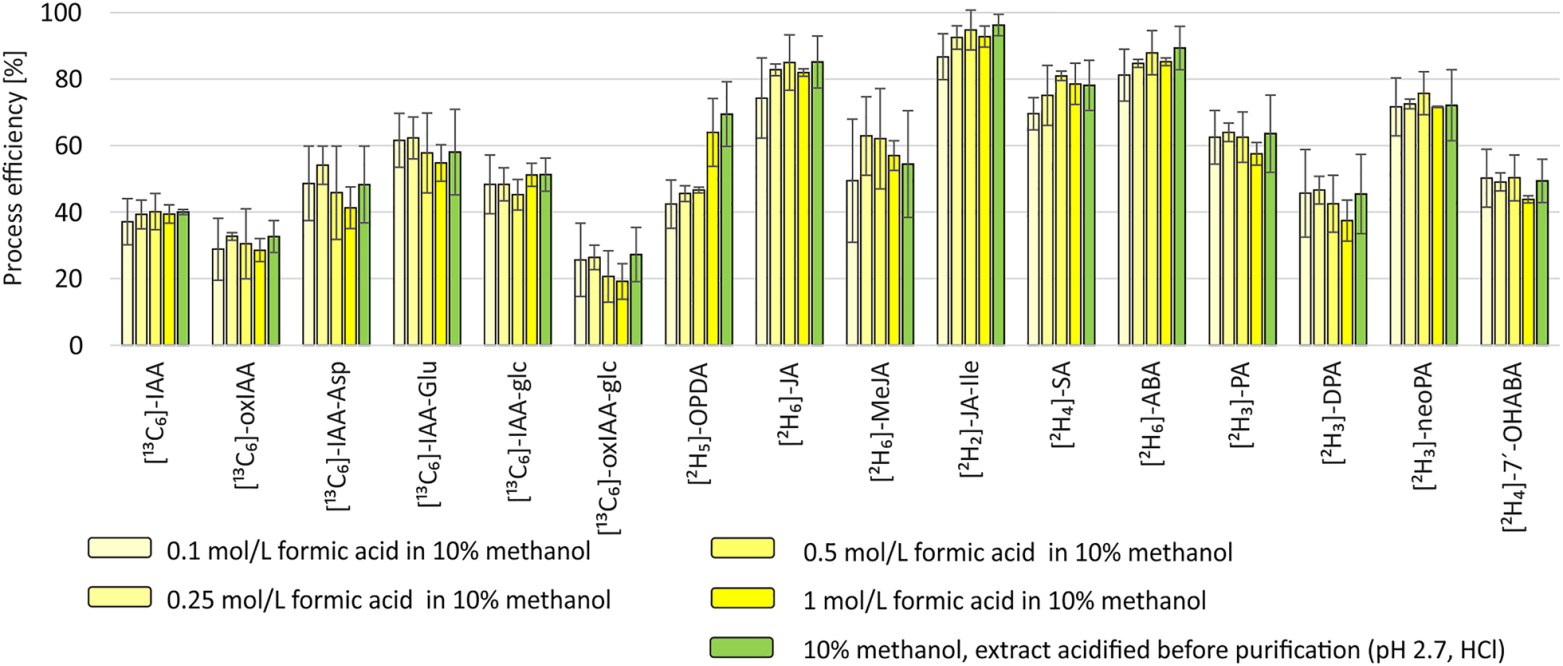
The impact of acidification on process efficiency (PE). PE (mean area of IS spike before extraction and purification / mean area of IS spike without extraction and purification injected in the same amount × 100) was estimated using 2 mg FW of 10-day-old *Arabidopsis thaliana* seedlings. Error bars represent ± SD, n = 4

The impact of sample acidification on the retention of standards on microSPE sorbent (SDB-XC/C18) was tested. Authentic standards (1 pmol) in 10% aqueous methanol and in 1 mol/L formic acid in 10% aqueous methanol were loaded on microSPE tips and processed as described in Fig. 2. The mean purification recovery of all phytohormones was 50% for standards loaded in 10% aqueous methanol and 64% for standards in 1 mol/L formic acid in 10% aqueous methanol. Acidification was beneficial for the retention and subsequent elution of all phytohormones except for *cis-*OPDA and OPC-8 (Additional file 1: Fig. S1). Next, the concentration of acid in the extraction solvent and the method of acidification were investigated. Process efficiency (PE) was assessed using 2 mg FW of *Arabidopsis thaliana* 10-day-old seedling matrix spiked with internal standard (IS; 1 pmol of all AUXs and JAs detected in ESI + ; 2 pmol for JAs detected in ESI-, ABAs and SA) extracted in 10% aqueous methanol containing different concentrations of formic acid (0.1–0.25–0.5–1 mol/L) and 10% aqueous methanol acidified to pH 2.7 (HCl) before purification. The mean PEs for extracts in 0.1–0.25–0.5–1 mol/L formic acid in 10% aqueous methanol and 10% aqueous methanol acidified to pH 2.7 (HCl) before purification were 55%, 59%, 58%, 57% and 60%, respectively. The results thus show no significant trend regardless of formic acid concentration or whether the sample was extracted in acidified solution or acidified before purification (Fig. 3).

A time-dependent increase in estimated *cis-*OPDA levels was previously reported in ground *Arabidopsis thaliana* plant material kept on ice [27]. This phenomenon was explained by possible residual activity of lipases releasing *cis-*OPDA from galactolipids—Arabidopsides [45]. Likewise to *cis-*OPDA, dn-OPDA is a constituent of the Arabidopsides [46]. When the influence of extraction approach on measured endogenous levels of phytohormones in *Arabidopsis thaliana* 10-day-old seedlings was studied (acidic extraction in 1 mol/L formic acid in 10% aqueous methanol and extraction in 10% aqueous methanol with pH adjusted before purification), endogenous *cis-*OPDA and dn-OPDA levels were found to be hugely dependent on the type of extraction (Additional file 1: Fig. S2). From these results we concluded that extraction in 1 mol/L formic acid can prevent potential enzyme activity and provide stable extraction conditions. On the other hand, the acidic conditions may decrease the stability of some analytes, especially JA or IAA conjugates with amino acids and glucose [34]. Regardless of stability, acidic conditions are crucial for the retention of the majority of analytes on reverse phase sorbent. The impact of analyte stability on precise quantification is compensated by appropriate internal standards, which are added to the plant material before extraction and get exposed to acidic conditions in the same way as the endogenous analytes.

To elute acidic plant hormones retained on the SPE reverse phase sorbent, a high percentage of organic solvent (e.g. 80% aqueous methanol) is utilised [33, 40, 43, 44]. In reverse phase chromatography of acidic phytohormones the gradients usually start at a low organic content. In order to achieve high quality of chromatographic peak shapes while injecting larger sample volumes (e.g. 10 μL), it is beneficial to include an evaporation step, which removes the excess organic solvent, and reconstitute the samples in a solvent with a low organic content similar to the composition of mobile phases at the beginning of the gradient [47]. This step also provides an opportunity to pre-concentrate the analytes. The optimum means of sample evaporation after elution (50 μL of 80% aqueous methanol) was investigated. Direct lyophilisation of 96-well plates was the most high-throughput approach available. The best recoveries were obtained using evaporation *in vacuo* (94%), followed by lyophilisation (93%); evaporation under a stream of nitrogen (89%) gave the lowest recovery (Additional file 1: Fig. S3). The most problematic compound was MeJA, in all probability due to its volatility [48].

### Method validation

The optimised high-throughput method was then validated. With the exception of OPC-8, the accuracies fell in the range 85–115% (Table 2). Despite the very similar structure and physico-chemical properties of OPC-8 and *cis-*OPDA, which differ only in the presence of a double bound on the cyclopentane ring (*cis-*OPDA), the internal standard, [^2^H_5_]-OPDA, was not able to fully compensate for losses during sample preparation, as can be seen from the different recoveries of these compounds (OPC-8 23% compared to *cis-*OPDA 43%) (Table 2). Precisions were lower than 15% for all analytes (Table 2).

**Table 2:** Validation parameters

Analyte	Method accuracy / precision^a,b^					RE^b,c^, %	PE^b,c,d^, %	ME^b,c^, %
Spike	0.1 pmol	0.5 pmol	1.0 pmol	5.0 pmol	10.0 pmol			
IAA	88.02/5.15	93.55/2.17	105.00/1.83	114.94/1.32	114.04/2.54	105.23 ± 6.53	61.61 ± 7.08	62.21 ± 3.14
oxIAA	94.16/9.37	106.24/1.08	113.01/2.06	113.97/2.66	113.74/1.21	32.11 ± 4.85	39.38 ± 9.66	98.02 ± 0.97
IAA-Asp	90.85/6.00	97.81/4.11	102.30/3.75	112.48/2.82	110.10/2.20	40.02 ± 2.63	46.25 ± 14.26	90.82 ± 1.34
IAA-Glu	92.73/0.70	109.57/4.04	112.09/1.71	113.40/2.40	112.48/2.47	66.38 ± 5.49	75.65 ± 14.20	111.86 ± 2.38
IAA-glc	NC	105.37/9.55	100.37/1.74	105.25/14.48	106.74/11.55	52.11 ± 7.83	37.71 ± 7.89	64.11 ± 4.83
oxIAA-glc	103.95/5.91	110.18/1.78	89.80/2.87	92.13/0.48	92.50/2.23	21.87 ± 8.24	28.25 ± 12.74	114.27 ± 3.34
*cis-*OPDA	94.92/0.87	102.94/1.72	114.65/2.57	112.29/2.88	113.96/1.73	42.71 ± 15.20	55.29 ± 15.00	102.80 ± 16.40
OPC-8	NC	NC	72.38/7.51	64.15/11.98	62.94/11.8	23.33 ± 1.30	–	112.27 ± 9.56
OPC-6	NC	89.02/7.16	86.27/3.67	90.84/6.03	87.64/4.25	71.02 ± 14.11	–	99.59 ± 8.32
OPC-4	111.04/5.83	97.33/2.63	93.69/7.36	103.65/2.7	100.87/0.58	103.03 ± 6.78	–	77.40 ± 4.28
dn-OPDA	101.73/7.03	105.74/4.96	108.58/3.32	103.68/4.26	97.80/4.47	86.43 ± 7.96	–	76.40 ± 0.82
JA	89.30/1.38	108.26/1.87	111.87/0.92	114.77/3.25	113.74/2.09	99.63 ± 2.60	70.73 ± 11.17	79.13 ± 1.21
MeJA	85.63/4.50	100.14/2.40	111.67/2.21	114.85/0.84	114.40/1.54	79.32 ± 5.84	40.40 ± 10.69	39.56 ± 7.87
9,10-dhJA	86.61/2.54	97.66/3.57	102.26/1.93	114.96/1.82	114.81/0.83	101.32 ± 2.33	–	81.84 ± 3.36
11-/12-OHJA	112.15/9.14	87.40/5.35	85.25/1.46	85.07/4.43	85.18/2.74	54.65 ± 7.25	–	70.76 ± 3.70
JA-Val	90.66/4.48	85.38/1.03	93.01/1.01	89.40/4.77	85.66/4.77	101.71 ± 5.96	–	100.09 ± 1.21
Ja-Ile	85.28/2.75	94.01/3.96	109.18/0.63	114.86/2.07	113.61/3.25	92.39 ± 14.39	106.74 ± 10.44	111.27 ± 1.01
JA-Trp	NC	114.94/6.34	103.75/4.66	114.06/3.99	112.02/3.34	90.55 ± 7.99	–	103.73 ± 0.90
JA-Phe	99.06/2.83	89.18/3.08	105.49/3.90	112.06/2.98	111.16/4.16	87.20 ± 14.06	–	106.15 ± 1.81
SA	111.82/5.7	85.06/3.55	89.71/2.95	110.52/1.24	110.79/1.01	86.12 ± 6.98	62.84 ± 4.10	62.73 ± 2.57
ABA	85.89/1.73	114.81/2.25	114.98/2.19	112.09/3.47	113.61/2.05	83.58 ± 2.41	77.69 ± 12.72	81.49 ± 1.42
PA	99.81/4.01	112.12/6.21	114.6/0.92	112.97/1.55	109.87/2.96	79.54 ± 1.98	62.77 ± 27.34	86.47 ± 1.19
DPA	NC	103.70/6.61	93.87/3.85	100.43/2.05	106.91/1.44	33.70 ± 5.31	42.65 ± 9.28	110.19 ± 2.37
neoPA	90.64/1.55	108.63/1.72	111.65/2.31	112.84/1.86	114.97/0.20	79.65 ± 3.86	74.24 ± 2.84	99.67 ± 2.05
7′-OHABA	88.33/5.83	105.69/3.28	106.62/3.12	114.27/3.72	110.59/3.10	83.88 ± 3.91	86.43 ± 3.27	108.82 ± 2.25

NC not calculated

^a^estimated by spiking 2 mg FW of 10-day-old *Arabidopsis thaliana* seedlings with authentic standards at four concentration levels

^b^values are means ± SD (n = 4)

^c^estimated by spiking 2 mg FW of 10-day-old *Arabidopsis thaliana* seedlings with authentic or internal standards at a single concentration level

^d^assessed for internal standards labelled with stable isotopes

Matrix effects (ME) express the signal suppression (ME below 100%) or enhancement (ME above 100%) caused by constituents of the sample matrix co-eluting with analytes; this occurs frequently when using MS/MS detection [49]. The ME for 2 mg FW of 10-day-old *Arabidopsis thaliana* seedlings ranged from 40 to 114%. The most pronounced ME was for MeJA (40%). Rather than ion suppression, the result is a consequence of the known volatility of MeJA [48] manifested during sample evaporation/lyophilisation, as the calculation of ME and also PE is related to neat standard solution, which was not subjected to sample preparation including evaporation (lyophilisation).

The recovery (RE) represents the losses of analyte in the course of sample preparation processes [49]. In our experiment the recoveries ranged from 22 to 105%. The process efficiency (PE) reflects both the influence of sample loss during sample preparation (RE) and ME [49]. The PE values were 27–107%. The lowest RE and PE results were obtained for oxIAA-glc (22 and 28% respectively). The low stability of oxIAA-glc contributed to these results, since spiking before extraction/purification resulted in longer exposure to acidic conditions compared to spiking after extraction/purification or neat standard solution.

### Phytohormone profiles of plant species representatives

**Fig. 4:**
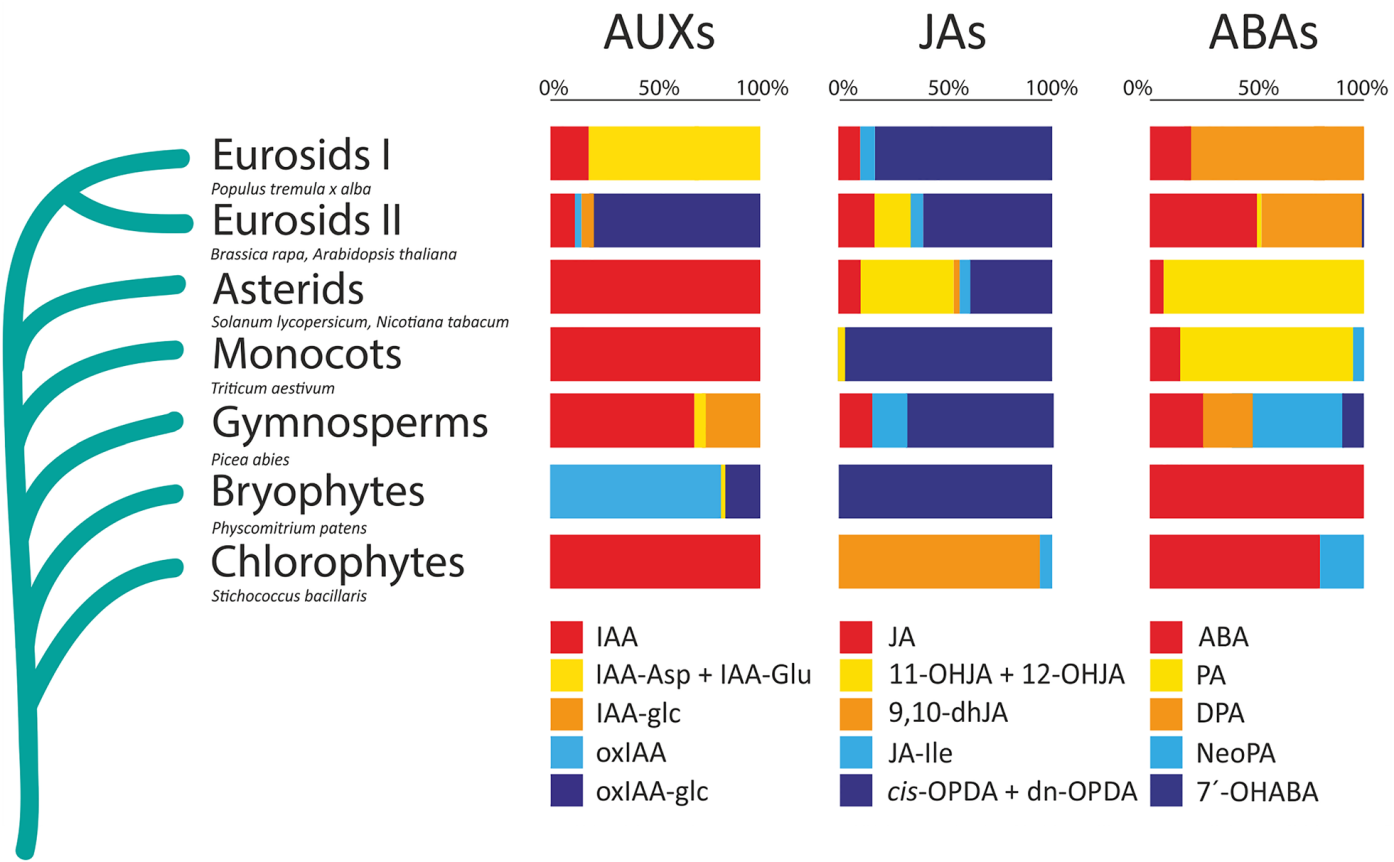
Schematic illustration of endogenous contents of AUXs, JAs and ABAs in selected species of land plants and algae assigned to groups. *Left panel*—Proportion of IAA, amide-linked IAA conjugates (IAA-Asp and IAA-Glu), IAA-glc, oxIAA and oxIAA-glc expressed as percentage of the total auxin pool. *Middle panel*–Proportion of JA, sum of 11-OHJA and 12-OHJA, 9,10-dhJA, JA-Ile, *cis-*OPDA and dn-OPDA expressed as percentage of the total jasmonate pool. *Right panel*–Proportion of ABA, PA, DPA, neoPA and 7´-OHABA expressed as percentage of the total abscisate pool. Results reported in Table 3 were used to draw this chart, when more species per group, the proportion was constructed based on the mean of results

The optimised high-throughput sample preparation protocol was applied to profile phytohormones in nine matrices of different plant species across the *Plantae* (Fig. 4). To evaluate the influence of sample amount on the ability of the method to quantify the acidic phytohormones, different amounts of these matrices (1–2–4–6–8 mg of FW and 0.1–0.2–0.4–0.6–0.8 mg of DW, *Stichococcus bacillaris*) were processed. The endogenous levels and the minimal sample weight necessary for detection and quantification of acidic phytohormones are listed in Table 3. Importantly, the sample weight and the levels of phytohormones found in that sample showed a linear relationship expressed as R^2^ ranging from 0.9820 to 0.9999 (Additional file 1: Table S1). Finally, we investigated how AUXs, JAs and ABAs contributed to the total pool in several distantly related species (Fig. 4).

**Table 3:** Phytohormone levels in different plant species

	*Populus tremula x alba*	*Arabidopsis thaliana*	*Brassica rapa*	*Solanum lycopersicum*	*Nicotiana tabacum*	*Triticum aestivum*	*Picea abies*	*Physcomitrium patens*	*Stichococcus bacillaris*
IAA	25.81 ± 1.66 (1 mg)	40.77 ± 4.12 (1 mg)	45.05 ± 2.20 (1 mg)	14.85 ± 1.70 (1 mg)	13.96 ± 1.16 (1 mg)	32.75 ± 2.10 (1 mg)	55.92 ± 3.14 (1 mg)	4.55 ± 0.31 (1 mg)	21.99 ± 2.65 (0.1 mg)
oxIAA	ND	142.20 ± 11.14 (1 mg)	ND	ND	ND	ND	ND	447.88 ± 25.92 (1 mg)	ND
IAA-Asp	69.33 ± 10.60 (6 mg)	18.66 ± 1.62 (4 mg)	ND	ND	ND	ND	ND	ND	ND
IAA-Glu	43.00 ± 3.28 (1 mg)	10.42 ± 1.34 (2 mg)	ND	ND	ND	ND	5.27 ± 0.57 (2 mg)	9.46 ± 1.06 (1 mg)	ND
IAA-glc	ND	352.64 ± 34.11 (1 mg)	ND	ND	ND	ND	23.38 ± 2.77 (4 mg)	ND	ND
oxIAA-glc	ND	3455.23 ± 260.74 (1 mg)	127.82 ± 11.48 (1 mg)	ND	ND	ND	ND	109.82 ± 14.99 (6 mg)	ND
*cis-*OPDA	18.25 ± 2.10 (1 mg)	18879.08 ± 2162.26 (1 mg)	143.46 ± 4.80 (1 mg)	156.30 ± 4.71 (1 mg)	ND	27691.98 ± 990.90 (1 mg)	137.06 ± 11.43 (1 mg)	74.75 ± 16.87 (1 mg)	ND
OPC-8	ND	ND	ND	ND	ND	ND	ND	ND	ND
OPC-6	ND	ND	ND	ND	ND	ND	ND	ND	ND
OPC-4	ND	842.59 ± 114.47 (1 mg)	ND	ND	ND	ND	ND	ND	ND
dn-OPDA	ND	11764.45 ± 1723.65 (1 mg)	ND	ND	ND	ND	ND	ND	ND
JA	2.30 ± 0.25 (1 mg)	268.28 ± 14.66 (1 mg)	222.15 ± 5.86 (1 mg)	23.42 ± 1.87 (1 mg)	8.69 ± 0.95 (1 mg)	4.58 ± 0.57 (1 mg)	21.91 ± 2.57 (1 mg)	ND	ND
MeJA	ND	ND	ND	ND	ND	ND	ND	ND	ND
9,10-dhJA	ND	ND	6.12 ± 0.74 (4 mg)	4.65 ± 0.28 (4 mg)	2.20 ± 0.14 (4 mg)	2.49 ± 0.30 (4 mg)	ND	ND	33.09 ± 3.18 (0.1 mg)
Sum of 11-OHJA and 12-OHJA	ND	23.22 ± 2.36 (2 mg)	292.07 ± 37.90 (1 mg)	ND	51.66 ± 5.40 (6 mg)	613.38 ± 48.63 (1 mg)	ND	ND	ND
JA-Val	ND	ND	ND	ND	ND	ND	ND	ND	ND
Ja-Ile	1.32 ± 0.17 (2 mg)	123.39 ± 10.12 (1 mg)	68.95 ± 3.46 (1 mg)	12.16 ± 1.23 (1 mg)	2.58 ± 0.25 (2 mg)	2.16 ± 0.22 (2 mg)	29.24 ± 3.45 (1 mg)	ND	1.04 ± 0.10 (0.4 mg)
JA-Trp	ND	ND	ND	ND	ND	ND	ND	ND	ND
JA-Phe	ND	ND	ND	ND	ND	ND	ND	ND	ND
SA	39.16 ± 2.49 (2 mg)	69.61 ± 6.81 (2 mg)	131.53 ± 69.14 (1 mg)	266.69 ± 54.80 (1 mg)	ND	152.06 ± 15.37 (1 mg)	40.50 ± 4.38 (2 mg)	183.86 ± 19.65 (1 mg)	103.38 ± 36.45 (0.2 mg)
ABA	6.49 ± 0.32 (1 mg)	1.26 ± 0.17 (2 mg)	16.65 ± 0.56 (1 mg)	701.63 ± 188.02 (1 mg)	6.98 ± 0.39 (1 mg)	4.10 ± 0.14 (1 mg)	89.34 ± 3.11 (1 mg)	0.91 ± 0.07 (4 mg)	2.38 ± 0.26 (1 mg)
PA	26.11 ± 3.16 (2 mg)	ND	37.47 ± 3.83 (1 mg)	4793.68 ± 294.18 (1 mg)	352.74 ± 21.66 (1 mg)	14.87 ± 1.17 (4 mg)	ND	ND	ND
DPA	ND	ND	1361.30 ± 134.40 (1 mg)	ND	ND	ND	65.09 ± 5.90 (6 mg)	ND	ND
neoPA	ND	ND	0.58 ± 0.07 (4 mg)	23.86 ± 1.58 (1 mg)	1.44 ± 0.21 (1 mg)	0.69 ± 0.03 (4 mg)	139.64 ± 11.52 (1 mg)	ND	0.65 ± 0.09 (0.4 mg)
7´-OHABA	ND	ND	20.23 ± 2.69 (2 mg)	11.90 ± 1.23 (4 mg)	ND	ND	43.62 ± 3.06 (1 mg)	ND	ND

Phytohormone levels are in pmol/g ± SD (the lowest sample amount in which the analyte was quantified) calculated from all amounts of samples in which the analyte was quantified. Results for 1 mg (n = 14), 2 mg (n = 12), 4 mg (n = 9), 6 mg (n = 6), 8 mg (n = 3), and for 0.1–0.8 mg of DW (*Stichococcus bacillaris*)

ND not detected

Free IAA was quantified in all the plant species analysed (Table 3). While free IAA was the only auxin detected in the chlorophyte *S. bacillaris*, in the monocot *T. aestivum* and in the two asterids, *N. tabacum* and *S. lycopersicum*, oxIAA followed by the glucosylesters of IAA and oxIAA were the most abundant auxin forms in the moss *Physcomitrium patens*, confirming previous findings reported for microalgae and bryophytes [50–52]. Among the seed plants, the proportion of ester conjugates in the total auxin pool was high in the conifer *P. abies* and in the two *Brassicaceae* species, *A. thaliana* and *B. rapa*. IAA-glc was the major glucosylester form in gymnosperms, while oxIAA-glc represented the majority of the total auxin pool in *Brassicaceae*, in accordance with published data [3, 10, 53]. The two amide conjugates of IAA, IAA-Asp and IAA-Glu, made up the majority of the total auxin pool in the woody eudicot hybrid poplar, confirming a previous finding reported in poplar where the IAA conjugate concentration was found to be higher than that of free IAA [54]. Interestingly, oxIAA was not detected in hybrid poplar leaves, while oxIAA was previously found to contribute to the asymmetric IAA distribution in bent roots of black poplar [55]. These differences in the auxin pool in poplar species might reflect the different tissues analyzed here (leaves, this study; roots, [55]) and/or species-specific metabolic pathways (hybrid poplar, this study; black poplar, [55]). Our results seem to corroborate the finding that universal elements exist among land plants that appear to form a metabolome in order to efficiently modulate auxin levels via conjugation and/or oxidation.

In jasmonate profiles, JA was detected in all the species analysed, with the exception of the moss *P. patens* and the chlorophyte *S. bacillaris*. Interestingly, the dihydro-oxylipin 9,10-dhJA and JA-Ile were the only JAs detected in the chlorophyte *S. bacillaris*, and to our knowledge this is the first report of a study where jasmonates were analyzed in this species. 9,10-dhJA was also found in fungi and plants, but descriptions of the presence of this dihydro-pentanone in plants are still limited. However, it has been reported that *cis-*OPDA was detected in the charophyte *Klebsormidium flaccidum*, but not in the charophyte *Chara braunii* (reviewed in [14]). *cis-*OPDA was the only jasmonate detected in the moss *P. patens* and this finding is in agreement with previous studies in the two bryophytes *P. patens* and *Marchantia polymorpha*, where JA and JA-Ile were not detected [56–58], whereas these metabolites could be found in a wide range of other bryophytes [51]. Literature on the distribution of JA and its derivatives in the lower plants, bryophytes and algae, appears to be contradictory and the presence or absence of jasmonates in bryophytes and algae requires further studies for confirmation [14]. Nonetheless, currently available evidence suggests that *cis-*OPDA is not only the precursor of JA biosynthesis but has distinct physiological roles from those of JA-Ile, acting through a JA-independent signalling pathway, and that its biosynthesis originated in the algal lineage, before the emergence of land plants [14, 25, 59, 60]. For gymnosperms, *cis-*OPDA was found to be the most abundant jasmonate in the conifer *P. abies*, followed by JA and JA-Ile (Fig. 4) and this is in agreement with findings described in [61]. In the grass *T. aestivum*, *cis-*OPDA and, to a lesser extent, 12-OHJA, were the two most abundant jasmonates detected (Fig. 4), while JA, JA-Ile and 9,10-dhJA were found only at very low concentrations (Table 3). With respect to the dicotyledonous species, JA and JA-Ile similarly contributed to the total jasmonate pool, whereas apparent dissimilarities were found for the other jasmonates profiled (Fig. 4). For instance, dn-OPDA was detected only in Arabidopsis samples and, interestingly, it was at a similar concentration to that of *cis-*OPDA (Table 3). The dihydro-pentanone 9,10-dhJA was found only in the two asterids and *B. rapa*, not in *A. thaliana* or hybrid poplar (Table 3).

ABA was detected in all the species analyzed. ABA, followed by neoPA, contributed the most to the total abscisate pool in the chlorophyte *S. bacillaris*, confirming the presence of ABA in this species (reviewed in [62]). In the bryophytes, ABA was the only abscisate found in the moss *P. patens*. However, various ABA metabolites were detected in several other bryophytes, as reported in [51]. The conifer *P. abies* seems to prefer to catabolise ABA through the formation of neoPA, as this metabolite was found to be more abundant than DPA and 7´-OHABA. This is in contrast with findings reported in [63] where the main hydroxylated ABA catabolite was PA, followed by 9′-hydroxyABA and DPA, whereas neoPA and 7′-OHABA were below detection limits. These differences might be due to the age of the plants analyzed (2 week-old, this study; 6 week-old, [63]) and/or on the growth conditions used (vermiculite-grown seedlings, this study; hydroponic-grown seedlings, [63]). Among the angiosperms, the grass and the asterids appear to preferentially catabolise ABA through the formation of PA, while hybrid poplar catabolises ABA through DPA formation (Fig. 4). ABA was the only abscisate detected in *A. thaliana*, whereas DPA was found to be the most abundant ABA catabolite, followed by PA, 7´-OHABA and neoPA in *B. rapa* (Table 3). It should be noted that the catabolic dynamics that regulate the ABA content in plants can be subject to significant changes upon stresses that trigger the ABA response.

## Conclusion

The acidic plant hormones are important regulators of plant processes, and comprehensive data on their profiles is often fundamental for plant biology research. A simple method for acidic plant hormone profiling comprising acidic extraction, high-throughput reverse phase in-tip microSPE sample purification using a 3D printed device and sensitive LC–MS/MS analysis was optimised and validated. The method allows simultaneous screening of 25 phytohormones and their precursors and metabolites and makes it possible to track their presence and the dynamics of their turnover in various species, requiring only units of mg of plant samples. Our protocol currently represents a fast and simple approach to phytohormone profiling that facilitates the processing of large sets of diverse plant samples.

## Methods

### Chemicals

Authentic and stable isotope labelled standards (Table 1) were provided from in-house standard library of JAs: *cis-*OPDA, OPC-8, OPC-6, OPC-4, dn-OPDA, JA, MeJA, 9,10-dhJA, 12-OHJA, JA-Val, JA-Ile, JA-Phe and [^2^H_5_]-OPDA, [^2^H_6_]-JA, [^2^H_2_]-( − )-JA-Ile were purchased from OlChemIm Ltd. (Olomouc, Czech Republic), JA-Trp was synthetised as described in [64], 11-OHJA was kindly provided by Dr. Otto Miersch, [^2^H_6_]-( ±)-MeJA was synthesized by acetyl chloride–methanol-d_4_ esterification; AUXs: IAA was purchased from Sigma Aldrich (St. Louis, MO, USA), oxIAA, IAA-Asp, IAA-Glu, IAA-glc, oxIAA-glc and [^13^C_6_]-IAA, [^13^C_6_]-IAA-Asp, [^13^C_6_]-IAA-Glu were purchased from OlChemIm Ltd. (Olomouc, Czech Republic), [^13^C_6_]-oxIAA was synthesized as described in [65], [^13^C_6_]-IAA-glc, [^13^C_6_]-oxIAA-glc were synthesized as described in [66, 67]; ABAs: ABA, PA, DPA, neoPA, 7′-OHABA and [^2^H_6_]-ABA, [^2^H_3_]-PA, [^2^H_3_]-DPA, [^2^H_3_]-neoPA, [^2^H_4_]-7′-OHABA were purchased from National Research Council Canada (Saskatoon, Canada); SAs: SA and [^2^H_4_]-SA were purchased from Sigma Aldrich (St. Louis, MO, USA). Methanol, acetonitrile, formic acid, all LC–MS grade, acetic acid of gradient grade and hydrochloric acid were purchased from Sigma-Aldrich (St. Luis, MO, USA). Purified Milli-Q water from a Simplicity 185 System was used for preparation of aqueous solutions.

### Plant material and growth conditions

*Arabidopsis thaliana —* seedlings of 10-day-old *Arabidopsis thaliana* ecotype ‘Col-0’ grown in vitro in Murashige and Skoog medium Petri dishes in a growth chamber at 20 °C, under long-day photoperiod (16 h light, 8 h dark) with a light intensity of 90 µmol photons m^−2^ s^−1^, were used for the optimisation of the extraction and purification protocol, method validation and determination of phytohormones in a series of matrices from different specimens.

*Populus tremula x alba – *the hybrid poplar seedlings were propagated vegetatively and grown in vitro in jars containing 20 mL of media (2.183 g/L Murashige and Skoog 1A media, 1 g/L glucose, and 8 g/L plant agar enriched with 112 mg/L Gamborg’s B5 vitamin mixture, L-Glutamine 20 mg/L, pantothenate 0.1 mg/L, L-cysteine chlorhydrate 0.1 mg/L and biotin 0.001 mg/L) under long-day conditions (16 h light at 22 °C, 8 h dark at 18 °C). The leaves from 90-day-old seedlings were cut at petiole and used in this study for phytohormone quantification.

*Brassica rapa — *leaves from 4-week-old *Brassica rapa* (cultivar ‘Pekinensis’) seedlings, germinated as described in [68] and cultivated in soil with vermiculite (2:1, v/v, respectively) in a growth chamber under long-day conditions at 21 °C, were used for phytohormone quantification.

*Solanum lycopersicum* — leaves from 8-week-old tomato seedlings (cultivar ‘Tornado’) cultivated in soil under environmentally controlled greenhouse conditions (natural photoperiod) during spring 2020 (from the middle of March to the middle of May) (Olomouc, Czech Republic), were used for phytohormone quantification.

*Nicotiana tabacum* — leaves from 8-week-old tobacco seedlings, cultivated in soil in a growth chamber under long-day conditions (16 h light at 20 °C, 8 h dark at 18 °C) were used for phytohormone quantification.

*Triticum aestivum *— leaves from 7-day-old seedlings of winter-type wheat (cultivar ‘Turandot’) were used in this study for phytohormone quantification. Seedlings were obtained from dormant seeds that were soaked for 5 h in tap water to germinate, sown into wet vermiculite, cultivated under long-day conditions at 21 °C, and irrigated every second day with 0.5 L of Hoagland’s nutrient solution [69].

*Picea abies *— 14-day-old spruce whole seedlings, grown in a growth chamber under long-day conditions (16 h light at 22 °C, 8 h dark at 18 °C) as described in [10], were used in this study for phytohormone quantification.

*Physcomitrium patens* — 3-week-old whole gametophores from *Physcomitrium patens* (Hedw.) Bruch & Schimp (ecotype ‘Gransden 2004′) wild type were used in this study for phytohormone quantification. The plants were grown axenically in 9-cm Petri dishes on Knop medium (100 mg/L Ca(NO_3_)_2_·4H_2_O, 25 mg/L KCl, 25 mg/L KH_2_PO_4_, 25 mg/L MgSO_4_·7H_2_O and 1.25 mg/L FeSO_4_·7H_2_O, pH 5.8). Knop medium was supplemented with 92.05 mg/L ammonium tartrate, 0.5 mg/L nicotinic acid, 0.125 mg/L *p*-amino benzoic acid, 2.5 mg/L thiamine HCl, trace-element solution (0.614 mg/L H_3_BO_3_, 0.389 mg/L MnCl_2_·4H_2_O, 0.059 mg/L NiCl_2_·6H_2_O, 0.055 mg/L CoCl_2_·6H_2_O, 0.055 mg/L CuSO_4_·5H_2_O, 0.055 mg/L ZnSO_4_·7H_2_O, 0.0386 mg/L Al_2_(SO_4_)_3_·18H_2_O, 0.028 mg/L KBr, 0.028 mg/L KI, 0.028 mg/L LiCl, 0.028 mg/L SnCl_2_·2H_2_O) and 200 mg/L glucose. Medium was solidified with 1.5% (w/v) plant agar. Plants were cultured under standard conditions in a growth chamber at 20 ± 1 °C, under a 16/8 h light/dark photoperiod with a light intensity of 90 µmol photons m^−2^ s^−1^. Plants were subcultured onto fresh medium every 3 weeks.

*Stichococcus bacillaris* — green algae were sampled in Nové Hrady (Czech Republic) in summer of 2013. Briefly, green algae were first pre-cultivated in batch cultures in shaking 100 mL Erlenmeyer flasks that were irradiated by 40 µmol photon m^−2^ s^−1^ from fluorescence tubes (Osram T8 Biolux). Late exponential growth was achieved within 5 days following the inoculation. Then, the cultures were transferred to glass tubes (V = 300 mL) in which the cultures were aerated and cultivated in a stronger light (120 µmol photon m^−2^ s^−1^) and in Bold´s basal medium [70]. Sample material was lyophilized, homogenized under liquid nitrogen and then used in this study for phytohormone quantification.

When harvested, all plant tissues were immediately frozen in liquid nitrogen and stored at − 80 °C.

### Sample extraction

Plant tissue was homogenised and powdered with a mortar and pestle under liquid nitrogen and reweighed into samples of approximately 10 mg FW or 1 mg of DW (*Stichococcus bacillaris*). The samples were extracted in cold 1 mol/L formic acid in 10% aqueous methanol so that 1 mL of the extraction solvent contained 10 mg FW or 1 mg DW. Internal standards were added to the samples to give 5 pmols of [^13^C_6_]-IAA, [^13^C_6_]-oxIAA, [^13^C_6_]-IAA-Asp, [^13^C_6_]-IAA-Glu, [^13^C_6_]-IAA-glc, [^13^C_6_]-oxIAA-glc, [^2^H_5_]-OPDA, [^2^H_2_]-( − )-JA-Ile, [^2^H_6_]-( ±)-MeJA and 10 pmols of [^2^H_6_]-JA, [^2^H_6_]-ABA, [^2^H_3_]-PA, [^2^H_3_]-DPA, [^2^H_3_]-neoPA, [^2^H_4_]-7′-OHABA, [^2^H_4_]-SA per 1 mL. To each of the samples, 4 ceria-stabilised zirconium oxide 2 mm beads (Retsch GmbH, Haan, Germany) were added, the samples were placed on an MM 400 mixer mill (Retsch GmbH, Haan, Germany) (29 Hz, 10 min, precooled holders), and centrifuged (25 200 g, 20 min, 8 °C), and 100 μL (1 mg of FW, 0.1 mg DW) or 200 μL (2 mg of FW, 0.2 mg of DW) of supernatant was loaded on a stagetip (Fig. 2A). The samples were handled at 8 °C in a CoolRack^®^ in a CoolBox^™^ (Biocision, Larkspur, CA, USA) in the course of the sample preparation steps.

### In-tip microSPE high-throughput purification

For in-tip microSPE columns, the stagetips [38] were self-assembled using ordinary pipette tips (2–200 μL, Eppendorf) and 3 layers of SDB-XC and C18 sorbent (3 M^™^ Empore^™^, St. Paul, MN, USA) similarly to the method published for auxin metabolite profiling [40] (Fig. 2B).

A 96-place tip-holder was projected in 123D Design (AutoDesk Inc., San Rafael, CA, USA) and printed by DeltiX (Trilab, Czech Republic) using polylactic acid filament. This holder was designed to be compatible with regular 96-well plates and a centrifuge rotor for 96-well plates (M-20, 75003624, Heraeus Megafuge 16 K centrifuge, Thermo Fisher Scientific, Waltham, MA, USA) (Fig. 2B). The design and dimensions of the device are provided in Additional file 1: Fig. S4. Depending on number of samples, the stagetips were accommodated in 3D-printed 96-place tip-holders adaptable to process different number of large sets of samples, up to 192 samples in one run.

The purification protocol was adopted from [40] with modifications (Fig. 2A). The stagetips, located in a centrifuge, were conditioned with 50 μL of acetone (320 g, 10 min, 8 °C), 50 μL of methanol (320 g, 10 min, 8 °C), and 50 μL of water (627 g, 15 min, 8 °C). 100 or 200 μL of sample extract (1742 g, 20 min; 8 °C) was applied, washed with 50 μL of 0.1% aqueous acetic acid in water (819 g, 20 min, 8 °C) and eluted with 80% aqueous methanol (1280 g, 20 min, 8 °C) into a 96-well plate suitable for direct injection onto an LC–MS/MS system. Apart from the sample loads, all liquids were applied with an eight-channel pipette (Fig. 2B). Finally, the eluents in the 96-well plate were lyophilized (Labconco, Kansas city, MO, USA) and the plate stored at − 20 °C until required for analysis. When evaporation techniques tested TurboVap LV system (Caliper Life Sciences, Hopkinton, MA, USA) for evaporation under gentle stream of nitrogen or Acid-Resistant CentriVaps benchtop concentrator (Labconco, Kansas city, MO, USA) for evaporation *in vacuo* were used.

### LC–MS/MS analysis

All analyses were carried out on an Agilent 6490 Triple Quadrupole LC/MS system coupled to a 1290 Infinity LC system (Agilent Technologies, Santa Clara, CA, USA). The data were processed in the MassHunter Quantitative software package version B.09.00 (Agilent Technologies, Santa Clara, CA, USA).

Purified and freeze-dried extracts were each dissolved in 40 μL of 20% aqueous acetonitrile and injected (10 μL) onto a Kinetex Evo C18 reverse phase column 2.1 ×150 mm, particle size 2.6 μm (Phenomenex, Torrance, CA, USA) protected by an inlet filter, using 10 mmol/L formic acid in water and methanol as mobile phases at a flow rate of 0.3 mL/min and a temperature of 50 °C. Gradient elution started at 2 min, going from 20 to 90% of methanol at 13.5 min, continuing within 0.5 min to 100% methanol. After 1 min of 100% methanol, the methanol content was decreased to 20% over the next 0.5 min and the column was equilibrated with 20% methanol for 3.5 min before the next injection.

The MS system was operated in dynamic multiple reaction monitoring mode in ESI positive and negative ionisation mode simultaneously (Table 1). The MS settings, multiple reaction monitoring transitions, and collision energies were optimised to previous standards. The nozzle voltage was set to 0 V, the capillary voltage to 2800/3000 V positive/negative mode, and the drying gas was at 130 °C with a flow rate of 14 L/min. The sheath gas was heated to 400 °C and its flow rate was set to 12 L/min.

### Method validation

The method was validated in terms of accuracy and precision at five concentration levels using 2 mg FW of 10-day-old *Arabidopsis thaliana* seedlings spiked with authentic (0.1, 0.5, 1.0, 5.0 and 10.0 pmol) and internal standards (1 pmol for all AUXs, JAs detected in ESI + , 2 pmol for JAs, ABAs, SA detected in ESI-) (Table 1) with four replicates. The accuracy was calculated using the determined levels of the analytes (pmols) with the endogenous levels subtracted divided by the nominal level of the spike (pmol) and expressed as a percentage of the nominal level. Precision was calculated as the relative standard deviation of determined levels (%). The PE, RE and ME were assessed as in [49] using 2 mg FW of plant matrix spiked before or after extraction and the purification procedure at one concentration level (4 pmol) with four replicates. Briefly, to evaluate PE, the mean peak area of the IS spiked before sample preparation was divided by the mean peak area of the neat solution of IS without any extraction and purification, expressed as a percentage. RE was calculated as the percentage of the mean peak rate of authentic standards spiked before and after the extraction and purification process. ME was calculated by dividing the mean peak area of authentic standards spiked after sample preparation by the peak area of the neat solution with the same amount of analyte. All validation samples were processed by the optimised extraction and purification protocol (Fig. 2).

To evaluate the influence of the method of drying the samples after purification, 50 μL aliquots of solutions of all authentic standards (at 0.5 pmol) in 80% aqueous methanol were processed using evaporation under a stream of nitrogen, *in vacuo* drying and lyophilisation, with four replicates of each. The dried samples were redissolved in 50 μL 20% aqueous acetonitrile mimicking the conditions immediately before LC–MS/MS analysis. The recovery was calculated by dividing the peak area of a dried standard by that of the original authentic standard solution, expressed as a percentage.

### Calibration and quantification

The contents of all samples were quantified using logarithmically transformed calibration curves constructed by plotting the responses of calibration standards (analyte peak area divided by IS area multiplied by IS concentration) against their known concentrations. The calibrations spanned from six to three orders of magnitude (Table 1).

### Amount of plant matrix required for phytohormone profiling

The weight of sample necessary for quantification of the analytes was evaluated using 1–2–4–6–8 mg FW or 0.1–0.2–0.4–0.6–0.8 mg DW of plant matrix. Either 1 or 2 mg FW (0.1 or 0.2 mg of DW) plant matrix were loaded on a stagetip, and eluates of 2 mg (0.2 mg of DW) were combined to obtain samples of 4 to 8 mg FW (0.4–0.8 mg DW). Each sample amount was analysed in either triplicate, or, for 1 mg FW (0.1 mg DW), in duplicate. The mean concentration of each analyte was calculated from all samples in which the analyte was quantified, e.g. an analyte quantified at 1 mg (n = 2) was also quantified at 2 mg (n = 3), 4 mg (n = 3), 6 mg (n = 3) and 8 mg (n = 3), giving a total of 14 samples (n = 14).

## Supplementary Information

**Additional file 1:** **Fig. S1.** Purification recoveries of standards in acidified and non-acidified solution. Calculated as mean area for standard solution (1 pmol) processed by microSPE divided by the area for the corresponding amount of standard without microSPE × 100. Error bars represent ± SD, n = 3. **Fig. S2.** Endogenous levels of dn-OPDA and *cis-*OPDA found in 10-day-old *Arabidopsis thaliana* seedlings using different extraction conditions over time. Error bars represent ± SD, n = 4. **Fig. S3.** Influence of the method of drying samples after purification using evaporation under a stream of nitrogen, *in vacuo* drying and lyophilisation, expressed as recovery after evaporation. Calculated as mean peak area for dried and re-dissolved standard divided by area for the standard solution at the same concentration without evaporation, expressed as a percentage. Error bars represent ± SD, n = 4. **Fig. S4.** Design and dimensions of 3D printed 96-place microSPE holder. **Table S1.** Range of sample weights (1–2–4–6–8 mg of FW or 0.1–0.2–0.4–0.6–0.8 mg of DW) in which the analyte was quantified, R^2^ of regression of sample weight and analyte level found in sample.

## Data Availability

The data used for this manuscript are available from the corresponding author on request.

## Abbreviations

7´-OHABA: 7′-Hydroxyabscisic acid
9,10-dhJA: 9,10-Dihydrojasmonic acid
11-OHJA: 11-Hydroxyjasmonic acid
12-OHJA: 12-Hydroxyjasmonic acid
ABA: Abscisic acid
ABAs: Abscisates
AUXs: Auxins
*cis*-OPDA: *cis*-( +)-12-Oxo-phytodienoic acid
dn-OPDA: Dinor-oxo-phytodienoic acid
DPA: Dihydrophaseic acid
DW: Dry weight
ESI: Electrospray ionisation
FW: Fresh weight
IAA: Indole-3-acetic acid
IAA-Asp: Indole-3-acetyl aspartate
IAA-Glu: Indole-3-acetyl glutamate
IAA-glc: Indole-3-acetyl-1-*O*-ß-D-glucose
IS: Internal standard
JA: Jasmonic acid
JA-Ile: Jasmonyl-isoleucine
JAs: Jasmonates
LC: Liquid chromatography
ME: Matrix effects
MeJA: Methyl jasmonate
MS: Mass spectrometry
MS/MS: Tandem mass spectrometry
neoPA: Neophaseic acid
oxIAA: 2-Oxindole-3-acetic acid
oxIAA-glc: 2-Oxindole-3-acetyl-1-*O*-ß-D-glucose
OPC-4: (*Z*)-4-[3-oxo-2-(pent-2-enyl)cyclopentyl]butanoic acid
OPC-6: (*Z*)-6-[3-Oxo-2-(pent-2-enyl)cyclopentyl]hexanoic acid
OPC-8: (*Z*)-8-[3-Oxo-2-(pent-2-enyl)cyclopentyl]octanoic acid
PA: Phaseic acid
PE: Process efficiency
RE: Recovery
SA: Salicylic acid
SAs: Salicylates
SD: Standard deviation
SPE: Solid phase extraction
