# Supplementary material for: High-throughput interspecies profiling of acidic plant hormones using miniaturised sample processing
Source: Plant Methods. 2022 Nov 16;18:122. doi: 10.1186/s13007-022-00954-3 (PMC9670418; doi:10.1186/s13007-022-00954-3)

Additional file 1: Figure S1. Purification recoveries of standards in acidified and non-acidified solution. Calculated as mean area for standard solution (1 pmol) processed by microSPE divided by the area for the corresponding amount of standard without microSPE * 100. Error bars represent ± SD, n = 3.

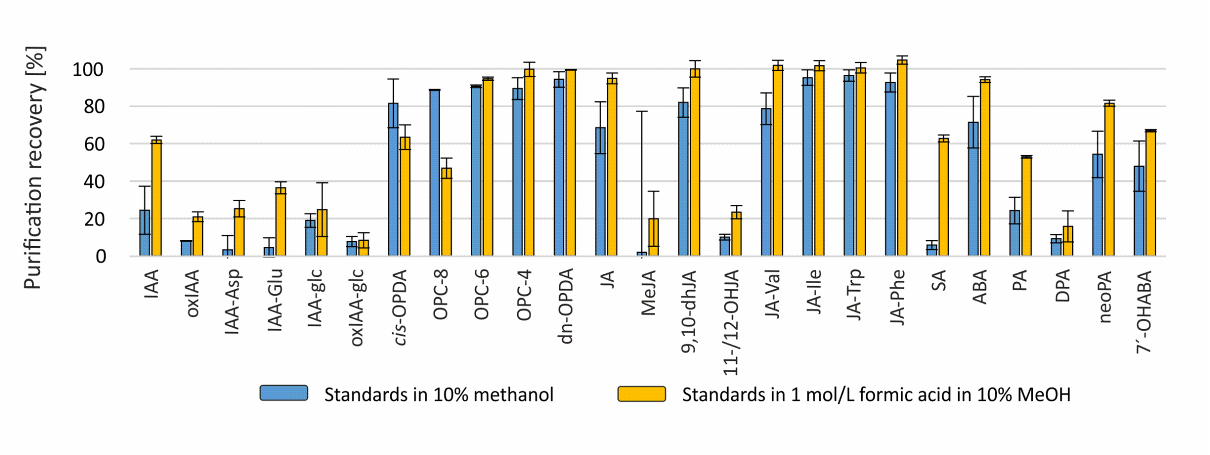

Additional file 1: Figure S2. Endogenous levels of dn-OPDA and *cis*-OPDA found in 10-day-old *Arabidopsis thaliana* seedlings using different extraction conditions over time. Error bars represent ± SD, n = 4.

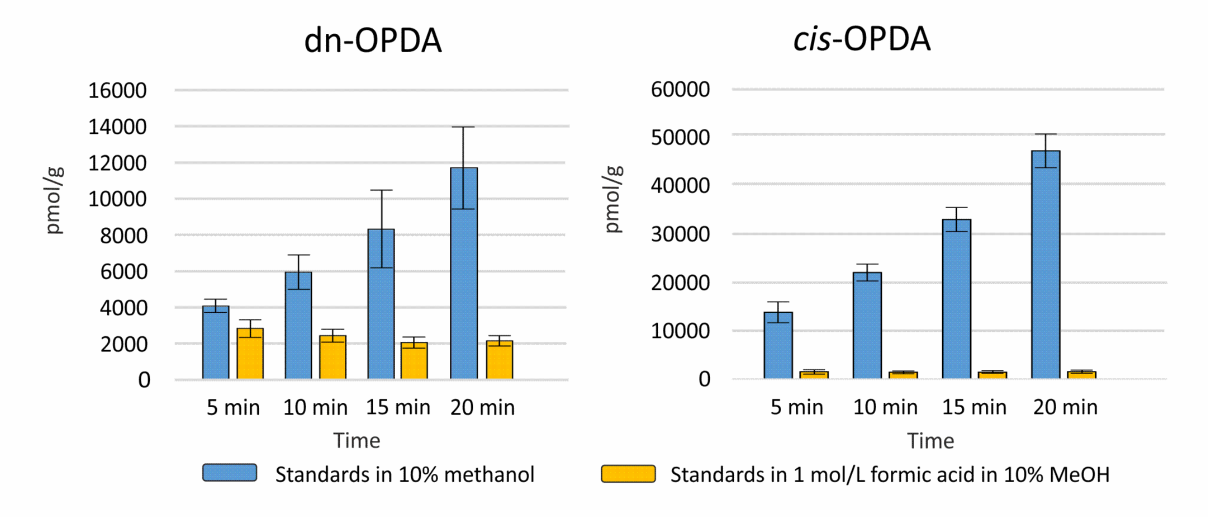

Additional file 1: Figure S3. Influence of the method of drying samples after purification using evaporation under a stream of nitrogen, *in vacuo* drying and lyophilisation, expressed as recovery after evaporation. Calculated as mean peak area for dried and re-dissolved standard divided by area for the standard solution at the same concentration without evaporation, expressed as a percentage. Error bars represent ± SD, n = 4.

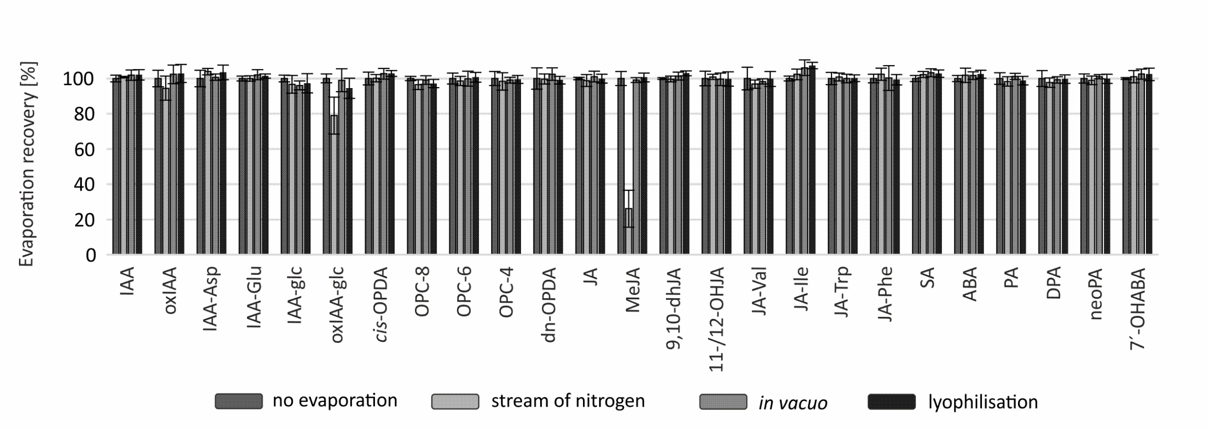

Additional file 1: Table S1. Range of sample weights (1 – 2 – 4 – 6 – 8 mg of FW or 0.1 – 0.2 – 0.4 – 0.6 – 0.8 mg of DW) in which the analyte was quantified, R2** of regression of sample weight and analyte level found in sample.

	Populus tremula x alba (FW)	*Arabidopsis thaliana* (FW)	*Brassica rapa* (FW)	*Solanum Lycopersicum* (FW)	*Nicotiana tabacum* (FW)	*Triticum aestivum* (FW)	*Picea abies* (FW)	*Physcomitrium patens* (FW)	*Stichococcus bacillaris* (DW)
IAA	1 - 8 mg, 0.9980	1 – 8 mg, 0.9962	1 - 8 mg, 0.9997	1 -8 mg, 0.9962	1 – 8 mg, 0.9968	1 – 8 mg, 0.9989	1 – 8 mg, 0.9996	4 - 8 mg, 0.9949	0.1 – 0.8 mg, 0.9968
oxIAA	ND	1 – 8 mg, 0.9992	ND	ND	ND	ND	ND	1 – 8 mg, 0.9996	ND
IAA-Asp	6 - 8 mg	4 – 8 mg, 0.9985	ND	ND	ND	ND	ND	ND	ND
IAA-Glu	1 – 8 mg, 0.9978	2 – 8 mg, 0.9820	ND	ND	ND	ND	2 – 8 mg, 0.9864	1 – 8 mg, 0.9985	ND
IAA-glc	ND	1 – 8 mg, 0.9993	ND	ND	ND	ND	4 – 8 mg, 0.9999	ND	ND
oxIAA-glc	ND	1 – 8 mg, 0.9993	1 – 8 mg, 0.9927	ND	ND	ND	ND	6 – 8 mg	ND
cis-OPDA	1 – 8 mg, 0.999	1 – 8 mg, 0.9983	1 – 8 mg, 0.9988	1 – 8 mg, 0.9996	ND	1 – 8 mg, 0.9943	1 – 8 mg, 0.9963	1 – 8 mg, 0.9991	ND
OPC-8	ND	ND	ND	ND	ND	ND	ND	ND	ND
OPC-6	ND	ND	ND	ND	ND	ND	ND	ND	ND
OPC-4	ND	1 – 8 mg, 0.9967	ND	ND	ND	ND	ND	ND	ND
dn-OPDA	ND	1 – 8 mg, 0.9988	ND	ND	ND	ND	ND	ND	ND
JA	1 – 8 mg, 0.9936	1 – 8 mg, 0.9987	1 - 8 mg, 0.9993	1 – 8 mg, 0.9968	1 – 8 mg, 0.9965	1 – 8 mg, 0.9997	1 – 8 mg, 0.9969	ND	ND
MeJA	ND	ND	ND	ND	ND	ND	ND	ND	ND
9,10-dhJA	ND	ND	4 – 8 mg, 0.9972	4 – 8 mg, 0.9903	4 – 8 mg, 0.9995	4 – 8 mg, 0.9998	ND	ND	0.1 – 0.8 mg, 0.9966
Sum of 11-OHJA and 12-OHJA	ND	2 – 8 mg, 0.9904	1 – 8 mg, 0.9948	ND	6 – 8 mg	1 – 8 mg, 0.9959	ND	ND	
JA-Val	ND	ND	ND	ND	ND	ND	ND	ND	ND
Ja-Ile	2 – 8 mg, 0.9989	1 – 8 mg, 0.9979	1 – 8 mg, 0.9983	1 - 8 mg, 0.9979	2 – 8 mg, 0.9955	2 – 8 mg, 0.9950	1 – 8 mg, 0.9979	ND	0.4 – 0.8 mg, 0.9998
JA-Trp	ND	ND	ND	ND	ND	ND	ND	ND	ND
JA-Phe	ND	ND	ND	ND	ND	ND	ND	ND	ND
SA	2 – 8 mg, 0.9999	2 – 8 mg, 0.9991	1 – 8 mg, 0.9981	1 – 8 mg, 0.9970	ND	1 – 8 mg, 0.9969	2 – 8 mg, 0.9973	1 – 8 mg, 0.9998	0.2 – 0.8 mg, 0.9937
ABA	1 – 8 mg, 0.9965	2 – 8 mg, 0.9994	1 – 8 mg, 0.9996	1 – 8 mg, 0.9986	1 – 8 mg, 0.9986	1 – 8 mg, 0.9998	1 – 8 mg, 0.9994	4 – 8 mg, 0.9996	0.1 – 0.8 mg, 0.9998
PA	2 – 8 mg, 0.9999	ND	1 – 8 mg, 0.9988	1 – 8 mg, 0.9999	1 – 8 mg, 0.9996	4 – 8 mg, 0.9973	ND	ND	ND
DPA	ND	ND	1 – 8 mg, 0.9927	ND	ND	ND	6 – 8 mg	ND	ND
neoPA	ND	ND	4 – 8 mg, 0.9979	1 – 8 mg, 0.9997	1 – 8 mg, 0.9996	4 – 8 mg, 0.9986	1 – 8 mg, 0.9990	ND	0.4 – 0.8 mg, 0.9904
7´-OHABA	ND	ND	2 – 8 mg, 0.9985	4 – 8 mg, 0.9962	ND	ND	1 – 8 mg, 0.9995	ND	ND

ND – not detected in any amount of plant matrix

Additional file 1: Figure S4. Design and dimensions of 3D printed 96-place microSPE holder.

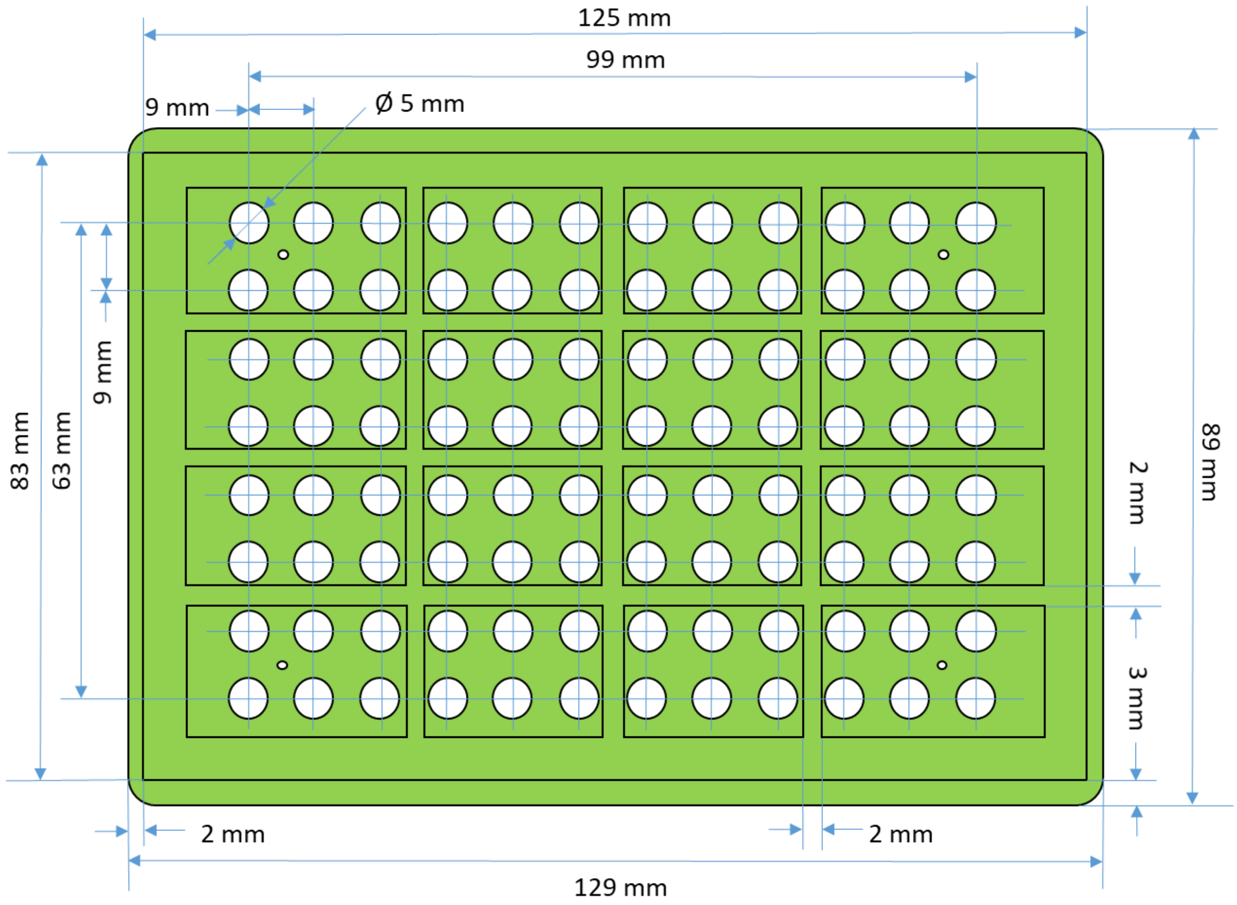

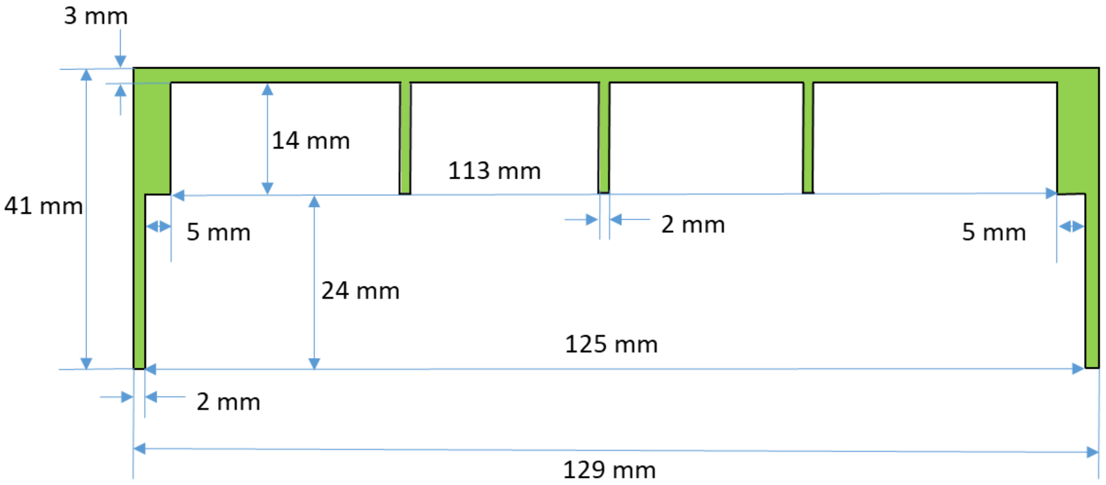

Supplement: Supplementary file 1 — Additional file 1: Fig. S1. Purification recoveries of standards in acidified and non-acidified solution. Calculated as mean area for standard solution (1 pmol) processed by microSPE divided by the area for the corresponding amount of standard without microSPE × 100. Error bars represent ± SD, n = 3. Fig. S2. Endogenous levels of dn-OPDA and cis-OPDA found in 10-day-old Arabidopsis thaliana seedlings using different extraction conditions over time. Error bars represent ± SD, n = 4. Fig. S3. Influence of the method of drying samples after purification using evaporation under a stream of nitrogen, in vacuo drying and lyophilisation, expressed as recovery after evaporation. Calculated as mean peak area for dried and re-dissolved standard divided by area for the standard solution at the same concentration without evaporation, expressed as a percentage. Error bars represent ± SD, n = 4. Fig. S4. Design and dimensions of 3D printed 96-place microSPE holder. Table S1. Range of sample weights (1–2–4–6–8 mg of FW or 0.1–0.2–0.4–0.6–0.8 mg of DW) in which the analyte was quantified, R2 of regression of sample weight and analyte level found in sample. [file 13007_2022_954_MOESM1_ESM.pdf]
